# Treatment with a Substance P Receptor Antagonist Is Neuroprotective in the Intrastriatal 6-Hydroxydopamine Model of Early Parkinson's Disease

**DOI:** 10.1371/journal.pone.0034138

**Published:** 2012-04-02

**Authors:** Emma Thornton, Robert Vink

**Affiliations:** Adelaide Centre for Neuroscience Research, School of Medical Sciences, The University of Adelaide, Adelaide, South Australia; Virginia Commonwealth University, United States of America

## Abstract

Neuroinflammation and blood brain barrier (BBB) dysfunction have been implicated in the pathogenesis of Parkinson's disease (PD). The neuropeptide substance P (SP) is an important mediator of both neuroinflammation and BBB dysfunction through its NK1 receptor in a process known as neurogenic inflammation. Increased SP content has previously been reported following 6-OHDA treatment *in vitro*, with the levels of SP correlating with cell death. The present study used an *in vivo* 6-OHDA lesion model to determine if dopaminergic degeneration was associated with increased SP in the substantia nigra and whether this degeneration could be prevented by using a SP, NK1 receptor antagonist. Unilateral, intrastriatal 6-OHDA lesions were induced and SP (10 µg/2 µL) or the NK1 receptor antagonists, N-acetyl-L-tryptophan (2 µL at 50 nM) or L-333,060 (2 µL at 100 nM), administered immediately after the neurotoxin. Nigral SP content was then determined using immunohistochemical and ELISA methods, neuroinflammation and barrier integrity was assessed using Iba-1, ED-1, GFAP and albumin immunohistochemistry, while dopaminergic cell loss was assessed with tyrosine hydroxylase immunohistochemistry. Motor function in all animals was assessed using the rotarod task. Intrastriatal 6-OHDA lesioning produced an early and sustained increase in ipsilateral nigral SP content, along with a breakdown of the BBB and activation of microglia and astrocytes. Further exacerbation of SP levels accelerated disease progression, whereas NK1 receptor antagonist treatment protected dopaminergic neurons, preserved barrier integrity, reduced neuroinflammation and significantly improved motor function. We propose that neurogenic inflammation contributes to dopaminergic degeneration in early experimental PD and demonstrate that an NK1 receptor antagonist may represent a novel neuroprotective therapy.

## Introduction

Parkinson's disease (PD) is the second most common neurodegenerative disorder affecting approximately 1% of the world's population over the age of 65 [1]. It is characterized by a progressive loss of dopaminergic neurons from the substantia nigra (SN), an integral part of the basal ganglia (BG). The BG is a group of nuclei that is primarily involved in the smooth execution of movement, and whose complex function requires that its two main signalling pathways be balanced. These pathways are kept in balance by the release of dopamine (DA) from dopaminergic neurons projecting from the SN to the striatum (caudaute nucleus and putamen). For proper function of the BG, a basal level of striatal DA release is required; therefore in PD, a loss of striatal DA causes motor symptoms such as bradykinesia, akinesia, rigidity and postural instability. Accordingly, current treatment for PD involves increasing striatal DA levels by either direct replacement with L-DOPA, by administering DA agonists or by reducing DA metabolism. However, these only provide symptomatic relief and do not target the cause of the dopaminergic cell loss. Moreover, while L-DOPA is highly effective in reducing motor symptoms for the first 5 to 10 years of use, continued use produces motor complications like dyskinesia and motor fluctuations [2]. Importantly, the progressive nature of cell loss in PD provides a window of opportunity in which a neuroprotective therapy could be administered to slow down or halt the progression of the disease. Unfortunately no known therapy exists to date.

Inflammatory processes and blood brain barrier (BBB) dysfunction have been associated with dopaminergic cell loss in PD. Activation of CNS immune cells such as microglia is observed in all animal models of PD, and in the SN of PD patients both on PET scans and at post-mortem [3], [4], [5]. Activated microglia damage cells by releasing pro-inflammatory cytokines, reactive oxygen species (ROS), nitric oxide (NO) and excitatory factors. An astrocytic response has also been observed in animal models of PD [3], [6], although the involvement of these cells in PD pathogenesis still remains controversial [7]. The loss of BBB integrity reported in PD is thought to also contribute to the progression of the disease [8], [9]. The barrier of the SN is known to be weaker than in other brain regions and therefore can be easily disrupted [10]. Moreover, dopaminergic neurons seem particularly vulnerable to BBB dysfunction, since induction of barrier breakdown leads to increased expression of apoptotic markers in DA neurons but not in hippocampal neurons [11].

An important mediator of both inflammation and increased BBB permeability in the CNS is the neuropeptide, substance P (SP). SP belongs to the tachykinin family, is widely found throughout the central and peripheral nervous systems, and preferentially binds to the NK1 tachykinin receptor, which like SP, is broadly distributed throughout the body. Accordingly, SP is involved in a diverse range of functions including smooth muscle contraction, transmission of sensory information, and nociception, amongst others (reviewed by [12]). SP is found in particularly high levels in the SN. Here, SP binds to NK1 receptors expressed on dopaminergic neurons, where internalisation of the SP/NK1 complex causes excitation and the release of DA into the striatum [13]. However, DA can also potentiate the release of SP within the nigra by binding to its D1 receptor located on striatal SP-containing medium spiny projection neurons [14]. Thus, SP and DA regulation is via a positive feedback mechanism.

Recently, 6-OHDA treatment of dopaminergic neurons *in vitro* produced an early and sustained increase in SP content, which significantly correlated with lactate dehydrogenase expression, a marker of cell death [15], suggesting that SP may play a role in dopaminergic cell. In the current study, we investigated whether SP may also be increased *in vivo* following induction of intrastriatal 6-OHDA lesions, a model of early PD, thereby initiating DA cell death by facilitating inflammation and BBB dysfunction.

## Results

**Figure 1:**
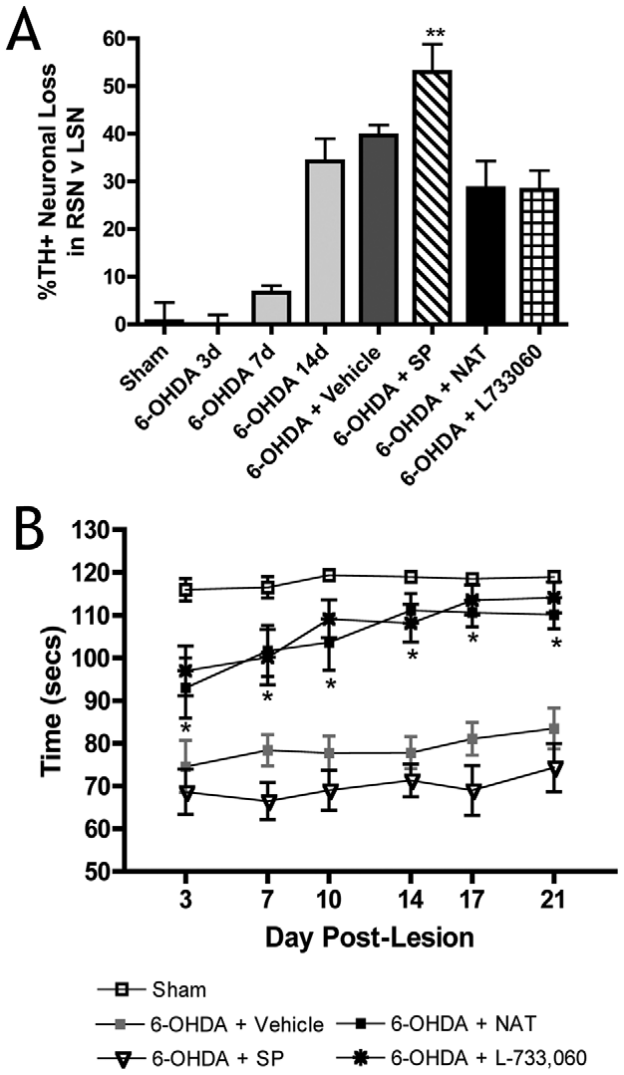
The effects of SP and NK1 antagonist treatment on nigral neuronal cell loss and motor function in rats following 6-OHDA intrastriatal lesions. (A) ipsilateral nigral dopaminergic neuronal loss as assessed by TH immunoreactivty; (B) motor function as assessed by an accelerating rotarod task.

Intrastriatal injections of 6-OHDA resulted in a progressive loss of TH positive neurons in the ipsilateral SN that was not apparent until day 7 post-lesion and was significant by day 14 (p<0.001; Figure 1A). By day 21, vehicle treated animals demonstrated further loss of these neurons. This loss was significantly exacerbated when SP was administered immediately after the 6-OHDA (p<0.01). Conversely, treatment with the NK1 receptor antagonists, NAT and L-733, 060, protected TH neurons, with these animals demonstrating a 28.5±5.8% and a 28.1±4.1% loss of neurons, respectively, compared to the 40.9±3.1% loss reported in vehicle treated animals.

The loss of ipsilateral TH positive nigral neurons produced a significant motor deficit as assessed by the rotarod task (Figure 1B). In contrast to sham animals who had normal motor function and could complete the 2 min task, vehicle treated animals were unable to learn the task and overall recorded a mean of 78.9±0.9 secs. SP treated animals displayed greater motor deficits as they performed only 69.8±1.1 secs on the rotarod task, whereas both NAT and L-733,060 treated animals performed significantly better than vehicle treated animals throughout the assessment period (p<0.05).

**Figure 2:**
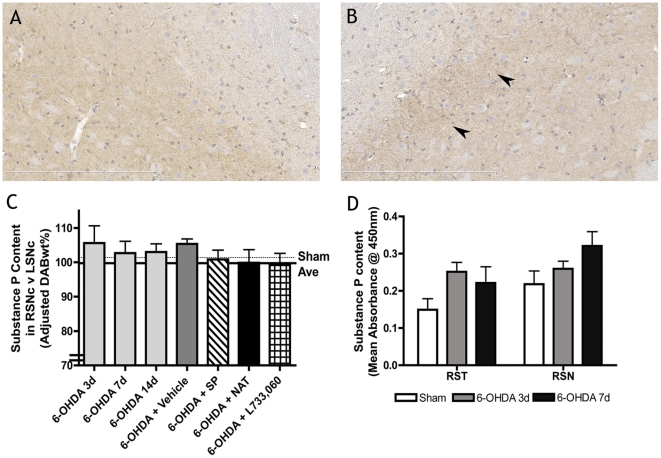
Changes in substance P content following intrastriatal 6-OHDA lesions. Substance P content in the ipsilateral SN (A) compared to the contralateral SN (B) is increased according to color deconvolution (C) and by an ELISA method (D). Arrows denote rise in SP. Bar = 300 µm.

To determine the mechanism by which NK1 antagonism protected DA neurons and improved motor function, SP content, BBB integrity and neuroinflammation was assessed. Intrastriatal 6-OHDA lesions produced an early and sustained increased in SP content in the ipsilateral SN compared to the contralateral SN (Figure 2A–C). Vehicle treated animals had greater SP content, whereas animals treated with SP or an NK1 antagonist had comparable SP content to shams. SP content was also measured at day 3 and 7 using an ELISA assay (Figure 2D). Again, an increase in SP content was recorded in the ipsilateral striatum and SN, with a greater rise seen in the striatum at day 3 and within the SN at day 7. Thus the day at which SP was maximal differed in each technique. This is likely due to ELISA assessing the entire SN and some of the surrounding tissue compared to color deconvolution, which primarily included the pars compacta region of the SN.

**Figure 3:**
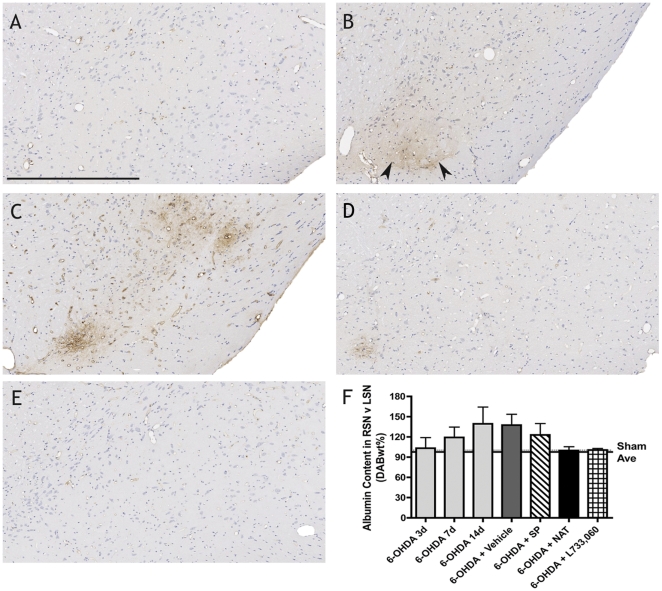
Changes in albumin extravasation following intrastriatal 6-OHDA lesions. Compared to sham animals (A), vehicle (B) and SP (C) treated animals demonstrate albumin immunoreactivity in the ipsilateral SN at day 21, whereas both NAT (D) and L-733,060 (E) treated animals resemble shams. Color deconvolution (F) confirms these trends. Arrows denote albumin. Bar = 500 µm.

6-OHDA intrastriatal lesions resulted in BBB breakdown and obvious albumin immunoreactivity in the ipsilateral nigra by day 7 post-lesion. Loss of barrier integrity was still apparent at day 21 as vehicle treated animals had marked albumin immunoreactivity (Figure 3). SP treated animals also demonstrated BBB dysfunction, however treatment with an NK1 antagonist preserved barrier integrity with both NAT and L-733,060 treated animals resembling sham animals.

**Figure 4:**
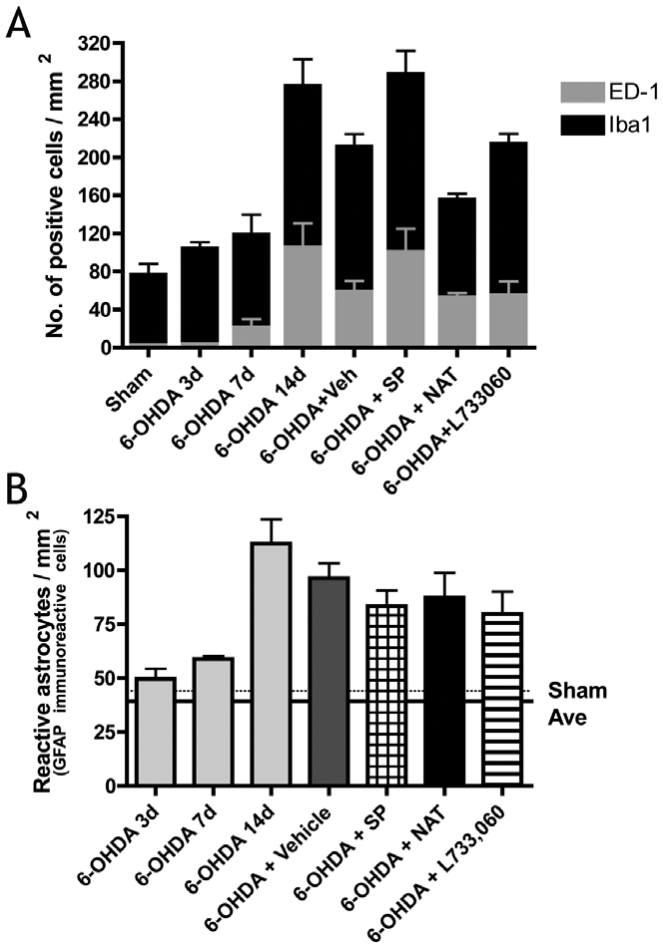
The effects of SP and NK1 antagonist treatment on neuroinflammation. Number of reactive (Iba-1 immunoreactive)/activated (ED-1 immunoreactive) microglia (A), and reactive astrocytes (B) in the ipsilateral SN following 6-OHDA intrastriatal lesions.

**Figure 5:**
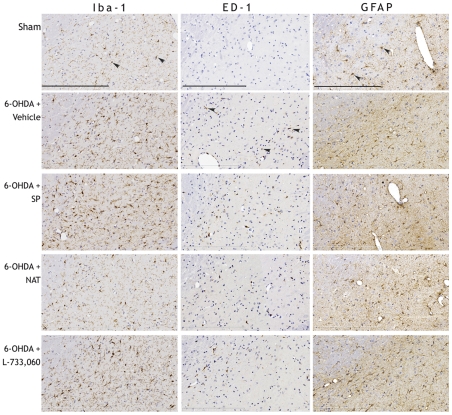
The effects of SP and NK1 antagonist treatment on Iba-1, ED-1 and GFAP immunoreactivty in the ipsilateral SN following intrastriatal 6-OHDA lesions at day 21. Arrows denote specified immunoreactivity. Bar = 400 µm.

Intrastriatal 6-OHDA lesions resulted in marked neuroinflammation (Figure 4 and 5). A small increase in Iba-1 positive reactive microglia was seen by day 3, and was maximal by day 14 at which point a significant increase in Iba-1 immunoreactive microglia was detected (p<0.01). Although the presence of ED-1 positive microglia was also maximal at day 14, the appearance of these cells was not seen until day 7 post-lesion. Vehicle treated animals had a significant increase in the number of both Iba-1 and ED-1 positive microglia compared to sham animals (p<0.01). SP treatment exacerbated the microglial response as these animals had a greater number of both Iba-1 and ED-1 immunoreactive microglia than vehicle treated animals, although this increase was not significant. Conversely, both NAT and a L-733,060 treated animals had an approximate 10% decrease in ED-1 immunoreactive microglia compared to vehicle treated animals. Despite this, only the NAT treated animals had a smaller number of Iba-1 positive microglia.

The presence of GFAP positive reactive astrocytes was similar to microglia. Maximal numbers of reactive astrocytes were recorded on day 14 post-lesion, with a highly significant increase still apparent in vehicle treated animals compared to sham animals (p<0.001). However, both SP and NK1 antagonist treatment resulted in a small decline in reactive astrocytes compared to vehicle treated animals, although a significantly greater number was still apparent compared to shams.

## Discussion

In the current study, we demonstrate that intrastriatal 6-OHDA lesions result in elevated SP production within the ipsilateral SN that remained above sham levels for 21 days. The increased SP levels were associated with increased BBB permeability, microglial and astrocyte activation, increased dopaminergic cell death and profound motor deficits. Moreover, treatment with additional SP at the time of 6-OHDA administration accelerated the progression of the disease, with animals displaying profound motor deficits and exacerbated dopaminergic cell death. In contrast, blocking the effects of SP with administration of an NK1 antagonist preserved barrier integrity, reduced inflammatory processes, attenuated 6-OHDA induced cell death and resulted in a significant improvement in motor function. To the best of our knowledge, this is the first demonstration that increased SP may play a critical role in early dopaminergic degeneration.

Although a potential role for SP in PD has been previously suggested, unlike in the current study, this was on the basis of decreased SP content and NK1 receptors within the BG in both clinical [16], [17], [18], [19], [20], [21] and experimental models of PD [22], [23]. These reports of SP decline were in post-mortem PD tissue and in models that replicate the late stages of the disease. It is likely that the observed loss of SP under these conditions is a secondary effect of dopaminergic degeneration, due to the interruption to the positive feedback regulation between SP and DA. Indeed, a 90% decrease in striatal DA has reportedly been required to deplete SP content within the SN [19]. In this study, nigral SP content was only decreased in the SP treated animals, which had significantly greater dopaminergic neuronal loss than any other group.

There are several possible mechanisms to account for the increase in SP seen in this study. During early dopaminergic degeneration, DA production is upregulated from surviving neurons in an attempt to attenuate the loss of striatal DA [24]. As mentioned, in the nigrostriatal pathway of the BG, SP and DA production are modulated by a positive feedback mechanism, whereby each neurotransmitter can potentiate the release of the other. Thus an increase in DA turnover may facilitate a localized increase in nigral SP. Another possible source of SP is other BG nuclei such as the globus pallidus (GP), which is also involved in the regulation of DA neurons, in part through SP containing projections from the internal segment of the GP to the SN [25]. Furthermore, the increase in nigral reactive astrocytes seen following intrastriatal 6-OHDA lesions can account for increased SP, as they are also known to produce SP [26].

This increase in SP is significant given that SP induces neurogenic inflammation, a neurally elicited inflammatory response caused by injury or infection, and involving the release of calcitonin-gene related peptide (CGRP) and SP from primary sensory nerve fibers surrounding blood vessels [27]. CGRP, a potent vasodilator, increases blood flow whereas SP, by binding to NK1 receptors on perivascular tissue, induces plasma extravasation, BBB breakdown and often genesis of edema [28]. Although this process has been well described in peripheral nervous system tissue, it has only recently been described following injury to the brain [27], [28], [29], [30]. Recently, a loss of BBB integrity has been implicated in the progression of PD [8], [9], [11].

SP induced breakdown of the BBB, as seen with albumin immunoreactivity, was noted in this study, with treatment with an NK1 antagonist preserving barrier integrity. Even a small breakdown in the BBB can be harmful to neurons by permitting an influx of peripheral immune cells, such as T-lymphocytes, blood borne macrophages and neutrophils into the brain. Peripheral immune cells also secrete pro-inflammatory cytokines, and once they enter the brain, they cannot only directly injure dopaminergic neurons, they can also activate resident microglia and astrocytes to indirectly contribute to cell loss. Indeed, CD4+ T-leukocytes have been implicated in MPTP induced DA cell death [31] and increased neutrophil infiltration has been associated with selective nigral dopaminergic degeneration in PD [32]. Prevention of the actions of SP through administration of an NK1 antagonist reduced levels of inflammation including a decrease in ED-1 immunoreactivity detected CD68 protein, a marker of both resident phagocytotic microglia and peripheral blood-borne macrophages [33]. We suggest that the early increase in SP content within the nigra may induce neurogenic inflammation and thus facilitate BBB breakdown and subsequent dopaminergic neuronal loss. This agrees with the reported actions of SP, which induces and augments many aspects of the inflammatory response including leukocyte activation, endothelial cell adhesion molecule expression and cytokine production.

Inflammatory processes are important mediators of 6-OHDA-induced cell death [34], [35]. Under normal conditions, the SN contains a large number of microglia as dopaminergic neurons are in a state of constant oxidative stress due to the production of ROS during DA metabolism [36]. Indeed, we found Iba-1 immunoreactive microglia were present within the SN of sham animals. Although microglia are vital to the control of immune and homeostatic functions within the brain, once activated they produce inflammatory cytokines such as IL-1, IL-6, and TNF-α, glutamate and quinolinic acid, superoxide radicals and NO [37]. A major source of ROS is microglial NADPH oxidase. In PD, both NADPH oxidase and ROS production are upregulated due to increased microglial activation [5]. ROS may cause apoptosis of neurons by inducing mitochondrial dysfunction and damaging lipids, proteins and DNA [38]. Indeed, ROS are thought to be major contributors to 6-OHDA-mediated cell death [39]. Notably, microglia express NK1 receptors and SP is considered to be a potent microglial activator [40], [41], [42].

In the current study, an increase in Iba-1 positive microglia was observed prior to TH neuronal loss, confirming that the appearance of activated microglia may proceed neurodegeneration [43]. Moreover, activation of microglia correlated with the degree of dopaminergic degeneration, as SP treated animals had both the greatest loss of DA neurons and the most nigral Iba-1 and ED-1 immunoreactive microglia. Conversely, animals treated with the NK1 antagonists had less Iba-1 immunoreactive microglia and a small reduction in the number of ED-1 positive microglia. Thus, inhibition of microglial activation using the SP, NK1 receptor antagonists may have contributed to the protection of dopaminergic neurons.

Astrocytes also express the NK1 receptor [44], and once activated by SP, NF-κβ translocates to the nucleus resulting in cytokine secretion [45]. An increase in GFAP positive reactive astrocytes was observed in the SN of all 6-OHDA groups. Reactive astrocytes may be beneficial by metabolizing excess cytosolic DA and through secretion of glial- and brain-derived neurotrophic factors and antioxidant enzymes [46]. Conversely, their secretion of pro-inflammatory cytokines may contribute to inflammatory processes and injury. In the current study, the effect astrocytes had on dopaminergic neurons is unclear as both SP and NK1 antagonist treated animals had a reduction in the number of these cells compared to vehicle treated animals. Nonetheless, due to SP's ability to induce proinflammatory cytokine secretion from astrocytes, we suggest that the reduction in reactive astrocytes by NK1 antagonists was beneficial and contributed to the preservation of dopaminergic neurons.

NK1 antagonism may also both directly reduce excitotoxic damage to DA terminals and neurons, or indirectly reduce excitotoxic damage by protecting dopaminergic neurons through other means and thus reduce subsequent subthalamic nucleus (STN) overactivity. Due to the functional arrangement of the BG, decreased striatal DA causes overactivity of the STN. The STN uses glutamate to send excitatory signals to the GP and SN to modulate the burst firing of dopamine neurons [47]. With no inhibition of the STN, excess glutamate will be released within the SN to bind to glutamate receptors located on dopaminergic neurons causing excitotoxic damage, a major contributor of cell death in PD [48]. Moreover, NMDA-evoked striatal glutamate release requires the activation of striatal NK1 and DA receptors [49].

In conclusion, SP is increased in the intrastriatal 6-OHDA model of early PD. This elevation in SP levels may contribute to the demise of dopaminergic neurons by modulating CNS inflammatory processes including the activation of microglia and astrocytes, as well as facilitating increased DA turnover and free radical production. This is the first study to implicate SP induced neurogenic inflammation in BBB breakdown and dopaminergic cell death in experimental early PD. Thus, NK1 antagonists may represent a novel therapeutic agent that may slow down progression of PD and prevent functional deficits in early PD.

## Materials and Methods

### Ethics Statement

All experimental protocols were conducted according to the guidelines established by the National Health and Medical Research Council of Australia and were approved by the animal ethics committees of the University of Adelaide and SA Pathology (approval number 65/05).

### Induction of PD

A total of 75 male, Sprague-Dawley rats weighing 250–300 g were group housed under a 12-hour light/dark cycle with access to food and water *ad libitum*. Animals were randomly assigned to sham (not lesioned) or 6-OHDA lesioning plus treatment with either an NK1 receptor antagonist (N-acetyl-L-tryptophan; NAT or L-733,060), SP, treatment vehicle (artificial CSF) or no treatment. The NK1 antagonist treated groups were administered either 2 µL of 50 nM NAT (Sigma) or 2 µL of 100 nM L-733,060 (Tocris) into the right lateral ventricle (stereotaxic co-ordinates: AP: −0.6 mm, ML: 1.5 mm relative to bregma and V: 3.5 mm from dura [50]) immediately following intrastriatal 6-hydroxydopamine-bromide (6-OHDA; Sigma) injections. The SP treated group was administered 2 µL of SP salt-acetate (5 µg/µL; Sigma) as described above for the NK1 antagonist groups. The NK1 receptor antagonist and SP doses were chosen on the basis of their known physiological effects in the nigra [51], [52]. The vehicle treated group received equal volume of vehicle (artificial CSF). Shams underwent the surgical procedure only without administration of the 6-OHDA.

The intrastriatal, 6-OHDA model of early PD has been previously described in detail elsewhere [53]. Briefly, animals were anesthetized with 3% Isoflurane (1.5 L/min O_2_), placed onto a stereotaxic device and two 0.7 mm burr holes then made over the right striatum at stereotaxic coordinates (1) AP: 0.5 mm, ML: 2.5 mm, and (2) AP: −0.5 mm, ML: 4.2 mm relative to bregma [50], [53]. Two µL of 6-OHDA (5 µg/µL) was then injected into the right striatum at 5.0 mm ventral from the dura in each of the two burr holes at a rate of 0.5 µL/min, with the needle then left in place for 2 min before being slowly retracted. Body temperature was maintained with a thermostatically controlled heating pad throughout the surgical procedures.

### Motor Outcome

Following induction of PD, animals were assessed for motor deficits on an accelerating rotarod. The rotarod is a sensitive test of motor deficits by testing general motor function including co-ordination and balance, and tests sensorimotor learning deficits in early stage PD [54]. The rotarod task has been described in detail elsewhere [55]. In this study, animals were not pre-trained but were tested at the same time each morning on days 3, 7, 10, 14, 17 and 21 post-lesion.

### Assessment of substance P, blood brain barrier and inflammation using immunohistochemistry

Following a predetermined survival period, animals were anesthetized with 5% Isoflurane, venously administered Heparin (5000 IU/mL) and transcardially perfused with 10% buffered formalin. The brain was cut into consecutive 2 mm coronal slices, processed and embedded in paraffin. The level containing the SN was cut into serial 5 µm sections that were processed as follows. Dopaminergic neurons were observed with tyrosine hydroxylase (TH) immunoreactivity (Chemicon; 1∶8000; citrate retrieval). Nigral SP protein content was determined using SP immunoreactivity (Santa Cruz; 1∶2000; EDTA retrieval). Iba-1 immunoreactivity (Wako; 1∶5000; citrate retrieval) and ED-1 immunoreactivity (AbD Serotec; 1∶400; citrate retrieval) was used to assess reactive and activated microglia. GFAP immunoreactivity (Dako; 1∶40000; citrate retrieval) assessed reactive astrocytes and BBB breakdown was observed using albumin immunoreactivity (Cooper Biomedical; 1∶20000; no retrieval).

All sections underwent a similar immunohistochemical procedure, with all antibodies incubated at room temperature and PBS washes applied between each antibody. Briefly, sections were de-waxed, dehydrated and placed in methanol with 30% hydrogen peroxide. Specified microwave antigen retrieval was performed as required and sections incubated for 45 min in 3% normal horse serum. Primary antibody was added overnight before specific biotinylated secondary antibody (Vector,1∶250) was added for 30 mins. Tertiary streptavidin peroxidase conjugate (SPC; Pierce, 1∶1000) was added for 1 hour and the immunocomplex visualised using 3,3′diaminobenzidine (DAB; Sigma) as a chromogen in the peroxidase reaction.

TH immunoreactive neurons were counted in the ipsilateral and contralateral SN at 20× magnification. The number of ipsilateral TH positive neurons was compared to contralateral neurons (internal control) to determine the percentage of ipsilateral dopaminergic cell loss. Iba-1 positive microglia, with intense cytoplasmic immunoreactivity and shortened processes, along with ED-1 immunoreactive microglia were counted in the ipsilateral SN at 20× magnification. The area of the SN was also determined to allow the number of reactive/activated microglia per mm^2^ to be determined. The number of reactive astrocytes was assessed in a similar manner to microglia with astrocytes counted that had upregulated GFAP immunoreactivty and shortened processes.

To non-subjectively estimate SP protein content and BBB integrity within the ipsilateral SN, all immunohistochemistry was subject to automated, color deconvolution analysis [54]. Briefly, SP and albumin immmunohistochemical sections were scanned using a Nanozoomer (Hamamatsu) before jpeg images at 20× magnification for SP and 10× magnification for albumin were exported from both the ipsilateral and contralateral SN. These jpeg images underwent color deconvolution, which separates the DAB color vector image from the hematoxylin color vector image. The amount of DAB precipitate (antigen content) was estimated using the histogram analysis of the DAB color image and a mathematical formula, which determined the percentage of weighted DAB (DABwt%) in the image. The weighting of the DAB is critical as it eliminates non-specific background staining whilst amplifying positive staining, and also gives a semi-quantitative estimate of intensity upon which statistical analyses can be performed [56]. As SP immunoreactivity can differ from batch to batch due to minor technical variations, each image was corrected using an internal control (interhemispheric leptomeniges), which is affected only by technique and not induction of PD. This allows an accurate assessment of the amount of SP content in the SN. Albumin immunoreactivity is unaffected between batches, however the amount of adjusted DABwt% within the ipsilateral SN was compared to contralateral SN (internal control).

### Semi-quantification of substance P using ELISA

At day 3 or 7 following intrastriatal 6-OHDA lesions, animals were re-anaesthetized with 5% Isoflurane (1.5 L/min 0_2_), decapitated and their brain removed. The striatum and midbrain were rapidly dissected out, the hemispheres divided and the cortex removed from the surrounding structures. Each brain region was weighed then homogenised in buffer containing 0.01% TritonX-100, DL-dithioreitol and protease inhibitors pepstatin A, aprotinin, leupeptin and phenylmethanesulfonyl fluoride. Protein content of the samples was estimated using a Biorad protein estimation assay, with samples diluted with tris-buffered saline (TBS) to 400 ng protein per 100 µl sample. An ELISA assay was used to determine the level of SP in the striatum and SN. Brain homogenate samples and control blank wells were loaded in triplicate and left overnight at 4°C. Blocking agent was added for 1 hour before 100 µL of SP primary antibody (Chemicon; 1∶1000) was incubated at 37°C for at least 1 hr. 100 µL of secondary anti-rabbit horseradish peroxidase conjugate (Rockford, 1∶500) antibody was incubated for 1 hr at 37°C before 100 µL/well of liquid substrate system 3,3′-5,5′-tetramethylbenzidine (Sigma) used to visualize SP content. TBS washes (3×) occurred between each step.

### Statistical Analysis

All data was analysed using an Analysis of Variance (ANOVA) followed by Neuman-Keuls multiple comparison posthoc tests (GraphPad Prism). Data is expressed as mean ± standard error of the mean (SEM).
